# Polarisome core component FgPea2 regulates FgBoi2-mediated polarized growth, pathogenicity and environmental stress in *Fusarium graminearum*

**DOI:** 10.1007/s44154-026-00300-w

**Published:** 2026-04-07

**Authors:** Xinrui Guo, Pengfang Li, Yunsi Xiao, Jingkun Liang, Cheng Zheng, Chenxuan Huang, Yakubu Saddeeq Abubakar, Zonghua Wang, Huawei Zheng, Jingwan Yan, Guo-dong Lu

**Affiliations:** 1 State Key Laboratory of Agricultural and Forestry Biosecurity, College of Plant Protection, Fujian Agriculture and Forestry University, Fuzhou, Fujian 350002 China; 2 Biotechnology Research Institute, Fujian Academy of Agricultural Sciences, Fuzhou, 350003 China; 3 College of Resources and Environment, Fujian Agriculture and Forestry University, Fuzhou, 350002 China; 4 Marine and Agricultural Biotechnology Laboratory, College of Geography and Oceanography, Minjiang University, Fuzhou, 350108 China

**Keywords:** *Fusarium graminearum*, Polarisome, FgBoi2, Polarized growth, Pathogenicity, Fungicide sensitivity

## Abstract

*Fusarium graminearum* is a devastating plant pathogenic fungus that is the predominant causal agent of Fusarium head blight. Our previous study showed that the polarisome core component FgPea2 regulates polarized growth, deoxynivalenol (DON) production and virulence of *F. graminearum*. To further investigate the regulatory network of FgPea2, FgPea2-GFP pull down was performed and the anillin-related protein FgBoi2 was identified. FgBoi2 interacts with FgPea2 and exhibits partial co-localization at the hyphal tips of *F. graminearum*. Interestingly, FgPea2 regulates the polarized localization of FgBoi2-GFP, suggesting that FgPea2 functions upstream of FgBoi2. Furthermore, deletion of *FgBOI2* resulted in a significant reduction in vegetative growth, conidiation, DON production and pathogenicity, as well as increased hyphal branching in *F. graminearum*. Structural analysis revealed that FgBoi2 contains SH3, SAM and PH domains. Further domain deletion experiments showed that the PH domain is crucial for vegetative growth, hyphae branching and polarized localization of FgBoi2. In addition, FgBoi2 negatively regulates the cell wall integrity and oxidative stress response pathways. FgPea2 and FgBoi2 modulate susceptibility to the fungicides tebuconazole, carbendazim, phenamacril and difenoconazole. In conclusion, this study shows that FgPea2 interacts with FgBoi2 and regulates FgBoi2-mediated polarized growth, stress tolerance, pathogenicity and fungicide resistance in* F. graminearum*.

**Supplementary Information:**

The online version contains supplementary material available at 10.1007/s44154-026-00300-w.

## Introduction

*Fusarium graminearum* is a devastating plant pathogenic fungus that causes Fusarium head blight (FHB) and leads to significant yield and quality losses in cereals worldwide (Moonjely et al. 2023). *F. graminearum* produces deoxynivalenol (DON), which is harmful to human health and serves as an important virulence factor for its host (Xu et al. 2022). Due to its devastating effect, *F. graminearum* has been included in the top 10 plant fungal pathogens (Dean et al. 2012). The fungus infects its hosts through vulnerable openings or the stomata (Bushnell et al. 2003), and also secrets effector proteins or mycotoxins to suppress the host’s immune response (Chen et al. 2019; Jiang et al. 2020; Xu et al. 2022; Shang et al. 2025). Many approaches, including genomics, genetic engineering, RNAi, novel fungicide chemistries, applied biocontrol, and consideration of the disease cycle, have been used for FHB management (Moonjely et al. 2023; Liu et al. 2025), e.g., application of dsRNA for FHB control of *F. graminearum* (Wu et al. 2025), application of valuable major QTL to improve FHB resistance in wheat (Zhang et al. 2024), AI-accelerated identification of novel antimicrobial peptides to inhibit *F. graminearum* (Ran et al. 2025). In addition, tebuconazole, carbendazim, phenamacril and difenoconazole are important *F. graminearum* fungicides (Wang et al. 2025; Xu et al. 2025).

The growth of hyphal tip is a key feature of filamentous fungi. The polarized growth of fungal hyphae requires that a supply of secretory vesicles be transported along cytoskeletal pathways to the site of cell expansion (Jones and Sudbery 2010). The spitzenkörper (SPK) was first identified as an iron hematoxylin body at the tip of *Coprinus sp.* hyphae and was thought to be associated with hyphal tip growth (Riquelme 2013). The polarisome is a multimeric complex within the polarity cap network localized at the SPK (Harris et al. 2005). Cosedimentation experiments demonstrate that Spa2, Pea2 and Bud6 form a large 12S multiprotein complex termed the polarisome (Knechtle et al. 2003), and recently, other potential polarisome components have been identified, including Aip5, Msb3 and Msb4 (Xie and Miao 2021). In yeast, the polarisome regulates microfilament formation at polarized growth sites (Sagot et al. 2002; Harris et al. 2005). Additionally, polarisome assembly mediates actin remodeling during polarized growth of yeast and other fungi (Xie and Miao 2021). Polarisome proteins play a critical role in reorganizing the actin cytoskeleton under low pH conditions (Motizuki and Xu 2009). Furthermore, the polarisome is important for the segregation and retrograde transport of protein aggregates (Liu et al. 2010). The phenotypes of yeast mutant strains lacking either *ScSPA2*, *ScPEA2* or *ScBUD6* are very similar, and have more spherical cells than the wild type; these mutants are defective in mating and bud site selection, and the cells are usually diploid (Gehrung and Snyder 1990; Sheu et al. 1998). In plant pathogenic fungi, components of the polarisome are important for polarized growth and virulence (Zheng et al. 2021; He et al. 2025).

The polarized growth of *F. graminearum* hyphae is critically for its infection processes (Zheng et al. 2021; Abubakar et al. 2023). However, the mechanism of *F. graminearum* hyphal polarized growth is not fully clarified. Our previous study showed that the polarisome core components FgSpa2, FgBud6 and FgPea2 are all critically required for the polarized growth, development and virulence of *F. graminearum* (Zheng et al. 2021). As a key component of the polarisome, Pea2 was first identify in yeast and it localizes to the sites of polarized growth and is required for efficient mating and bipolar budding (Chenevert et al. 1994; Valtz and Herskowitz 1996). Pea2 is important for Spa2 stability and correct localization (Valtz and Herskowitz 1996). HAP/ClpP expression affects the integrity of the actin cytoskeleton and causes failure in Spa2-dependent localization of Pea2 to the bud tip (Tessarz et al. 2009). Interaction of Pea2 with the ER-membrane-interacting protein Epo1 at the growing tip mediates the tethering between the polarisome and the endoplasmic reticulum (ER), and this facilitates the timely supply of membrane and cell wall materials (Xie and Miao 2021), indicating an important role of Pea2 in membrane and cell wall synthesis and assembly. We previously showed that the localization of FgPea2 is dependent on another polarisome component FgBud6 in *F. graminearum* (Zheng et al. 2021). However, Pea2 is still poorly studied in filamentous fungi such as *Neurospora crassa* (Lichius et al. 2012), *Aspergillus nidulans* (Virag and Harris Steven 2006) and *Aspergillus niger* (Meyer et al. 2008). Moreover, further exploration is needed to elucidate the cellular network of Pea2.

Boi1 and Boi2 are closely related yeast scaffolding proteins (Sulpizio et al. 2022). Boi2 functions in polarized growth and cytokinesis (Masgrau et al. 2017). It also has redundant function with Boi1 in promoting the fusion of secretory vesicles with the plasma membrane at the sites of polarized growth in yeast (Masgrau et al. 2017; Teyra et al. 2017). Despite this knowledge in yeast, the functions of Boi1/2 proteins are still largely unknown in other fungi, especially in plant pathogenic fungi. In this study, FgBoi2 was identified as a new interacting partner of FgPea2. FgBoi2 partially co-localizes with FgPea2 at the hyphal tip. We further found that FgPea2 regulates the polarized localization of FgBoi2, and deletion of *FgBOI2* causes significant reduction in the vegetative growth, conidiation, DON production and pathogenicity of *F. graminearum*. The PH domain of FgBoi2 was found to be indispensable for its function and correct localization. Moreover, FgBoi2 negatively regulates the fungal cell wall integrity and oxidative stress response pathways. Notably, the gene deletion mutants *∆Fgpea2* and *∆Fgboi2* exhibited remarkable resistance to the fungicides tebuconazole, carbendazim, phenamacril and difenoconazole.

## Results

### FgBoi2 interacts with the polarisome core component FgPea2

**Fig. 1:**
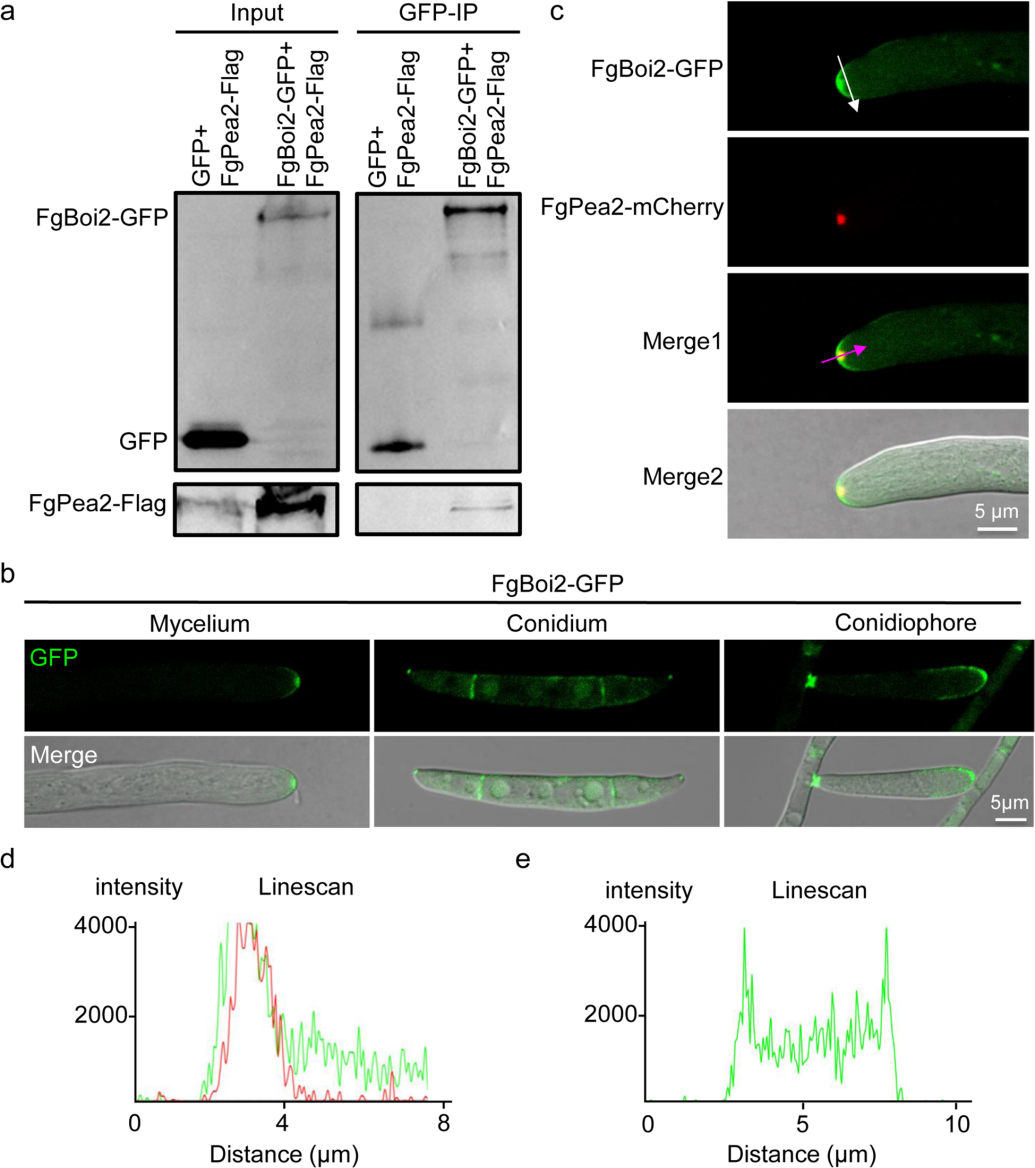
FgBoi2 interacts and partially co-localizes with polarisome core component FgPea2 in *Fusarium graminearum*. **a** Co-immunoprecipitation experiment indicating the interaction of FgBoi2 with FgPea2 in *F. graminearum*. **b** FgBoi2-GFP fusion protein localizes to the tip of mycelia, conidiophores and conidia. **c** FgBoi2-GFP partially co-localizes with FgPea2-mCherry in hyphal tips. **d** Line scan graphs showing the partial co-localization of FgBoi2-GFP with FgPea2-mCherry (purple arrow in Fig. 1c). **e** Line scan graphs showing the plasma membrane localization of FgBoi2-GFP (white arrow in Fig. 1c)

Our previous study has shown that the polarisome core component FgPea2 is localized at the hyphal tip and plays important roles in polarized growth, DON production and pathogenicity of *F. graminearum* (Zheng et al. 2021). To further investigate the regulatory mechanism of FgPea2, we performed pull down and mass spectrometry analyses of FgPea2-GFP. In these analyses, the anillin-related protein FGSG_10016 was identified. FGSG_10016 shows high similarity to the yeast budding protein Boi2, therefore we named it FgBoi2. To further determine the relationship between FgBoi2 and FgPea2, pFgBoi2-GFP and pFgPea2-flag vectors were generated and co-transformed into the protoplasts of PH-1. Then, Co-IP experiment was performed to further confirm the interaction of FgBoi2 with FgPea2. As shown in Fig. 1a, FgBoi2 positively interacts with FgPea2 in vivo. Interestingly, FgBoi2-GFP localizes to the tip of *F. graminearum* mycelia, conidiophores and conidia, supporting its polarisome-like localization in *F. graminearum* (Fig. 1b-c). Furthermore, a co-localization experiment showed that FgBoi2-GFP partially co-localizes with FgPea2-mCherry in hyphal tips (Fig. 1c), and the line scan graph further supports this result (Fig. 1d). In addition, we found that FgBoi2-GFP is partially localized in the plasma membrane of hyphal tips (Fig. 1b, c, e). Taken together, these results suggest that FgBoi2 has a polarisome-like localization and interacts and co-localizes with FgPea2 in *F. graminearum*.

Phylogenetic analysis showed that Boi2 is conserved in filamentous fungi, especially in *Fusarium oxysporum* and *Fusarium verticillioides* (Fig. S1a). Furthermore, to understand the physiological function of FgBoi2 in *F. graminearum*, the *FgBOI2* gene deletion mutants were obtained by a targeted gene replacement strategy (Fig. S1b). The *∆Fgboi2-1* and *∆Fgboi2*-*2* transformants were confirmed by Southern blot analysis, which showed a 3.6 kb band in the deletion mutants, and a 5.3 kb band in the wild type PH-1 strain (Fig. S1c).

### FgPea2 regulates the polarized localization of FgBoi2-GFP

**Fig. 2:**
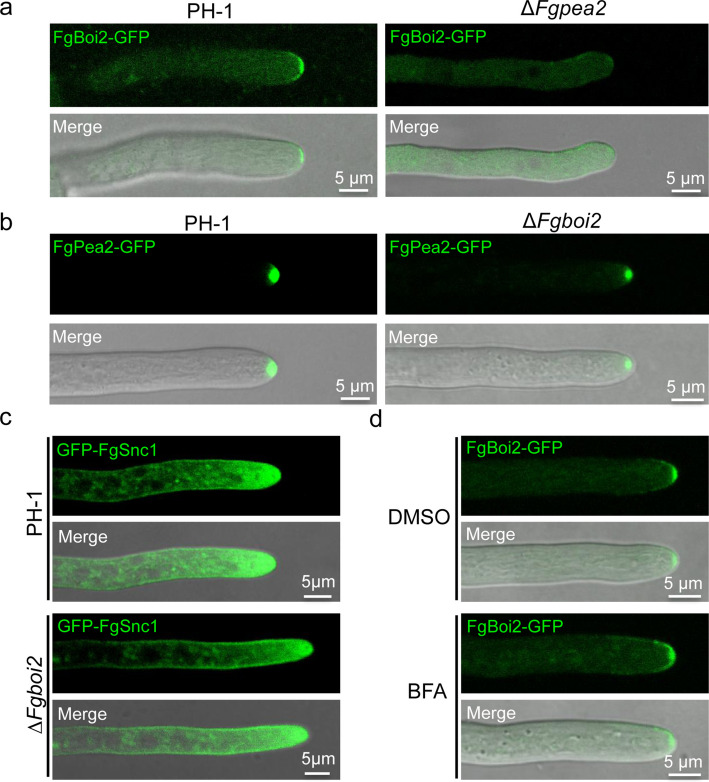
FgPea2 regulates the polarized localization of FgBoi2-GFP in *Fusarium graminearum*. **a** The localization of FgBoi2-GFP fusion protein in PH-1 and *FgPEA2* deletion mutant (*∆Fgpea2*). FgPea2 is required for the polarized localization of FgBoi2. **b** The localization of FgPea2-GFP fusion protein in PH-1 and *FgBOI2* deletion mutant (*∆Fgboi2*). FgBoi2 is dispensable for the polarized localization of FgPea2. **c** FgBoi2 is not required for GFP-FgSnc1-mediated polarized growth of *F. graminearum*. **d** Brefeldin A (BFA) does not affect the polarized localization or trafficking of FgBoi2-GFP in *F. graminearum*. The hyphae of FgBoi2-GFP strain were treated with 50 μg/mL BFA for 30 min

To further investigate the relationship between FgBoi2 and FgPea2, we constructed an FgBoi2-GFP fusion vector and transformed it into the protoplasts of *∆Fgpea2* mutant. As shown in Fig. 2a, FgBoi2-GFP localizes at the hyphal tip in the wild type. However, in the *∆Fgpea2* mutant, the fluorescence signal of FgBoi2-GFP was diffuse in the cytoplasm as well as around the plasma membrane of the hyphal tip. Furthermore, to investigate whether FgBoi2 regulates the polarized localization of FgPea2 in *F. graminearum*, we expressed FgPea2-GFP in *∆Fgboi2* mutant and found that FgPea2-GFP still displays polarisome-like localization in the hyphae of *∆Fgboi2* mutant (Fig. 2b). We further assayed the relative expression level of *FgBOI2* gene in the ∆*Fgpea2* mutant, and found that the relative expression level of *FgBOI2* gene in the ∆*Fgpea2* mutant was significantly decreased in comparison to that in wild type PH-1 (Fig. S2), suggesting that FgPea2 regulates the transcriptional level of *FgBOI2* gene. Taken together, these results suggest that FgPea2 is critical for the polarisome-like localization of FgBoi2 in hyphae tips.

SNARE (soluble N-ethylmaleimide-sensitive factor attachment receptor) proteins are known to mediate membrane fission and fusion of vesicles for effective delivery (Hong 2005). FgSnc1 is a v-SNARE protein that mediates polarized secretion and fusion of vesicles (Zheng et al. 2018). To explore the role of FgBoi2 in vesicles fusion with the plasma membrane, we transformed GFP-FgSnc1 vector into the protoplasts of PH-1 and *∆Fgboi2* mutant and subsequently observed its subcellular localization in the two strains. As shown in Fig. 2c, the GFP-FgSnc1 fusion protein is localized on the plasma membrane and also accumulates at the spitzenkörper (SPK) of the growing hyphal tip in both the wild type PH-1 and *∆Fgboi2* mutant strains, suggesting that FgBoi2 is dispensable for the biological functions of FgSnc1 in *F. graminearum*. Brefeldin A (BFA) inhibits the transport of proteins from the endoplasmic reticulum to the Golgi apparatus. To know whether the polarized localization of FgBoi2-GFP is depend on BFA, we treated the hyphae of FgBoi2-GFP strain with 50 μg/mL BFA. However, the signal of FgBoi2-GFP shows no significant difference compared to the DMSO control (Fig. 2d), suggesting that BFA does not affect the polarized localization or trafficking of FgBoi2-GFP in *F. graminearum*.

### FgBoi2 is required for the polarized growth of *F. graminearum*

**Fig. 3:**
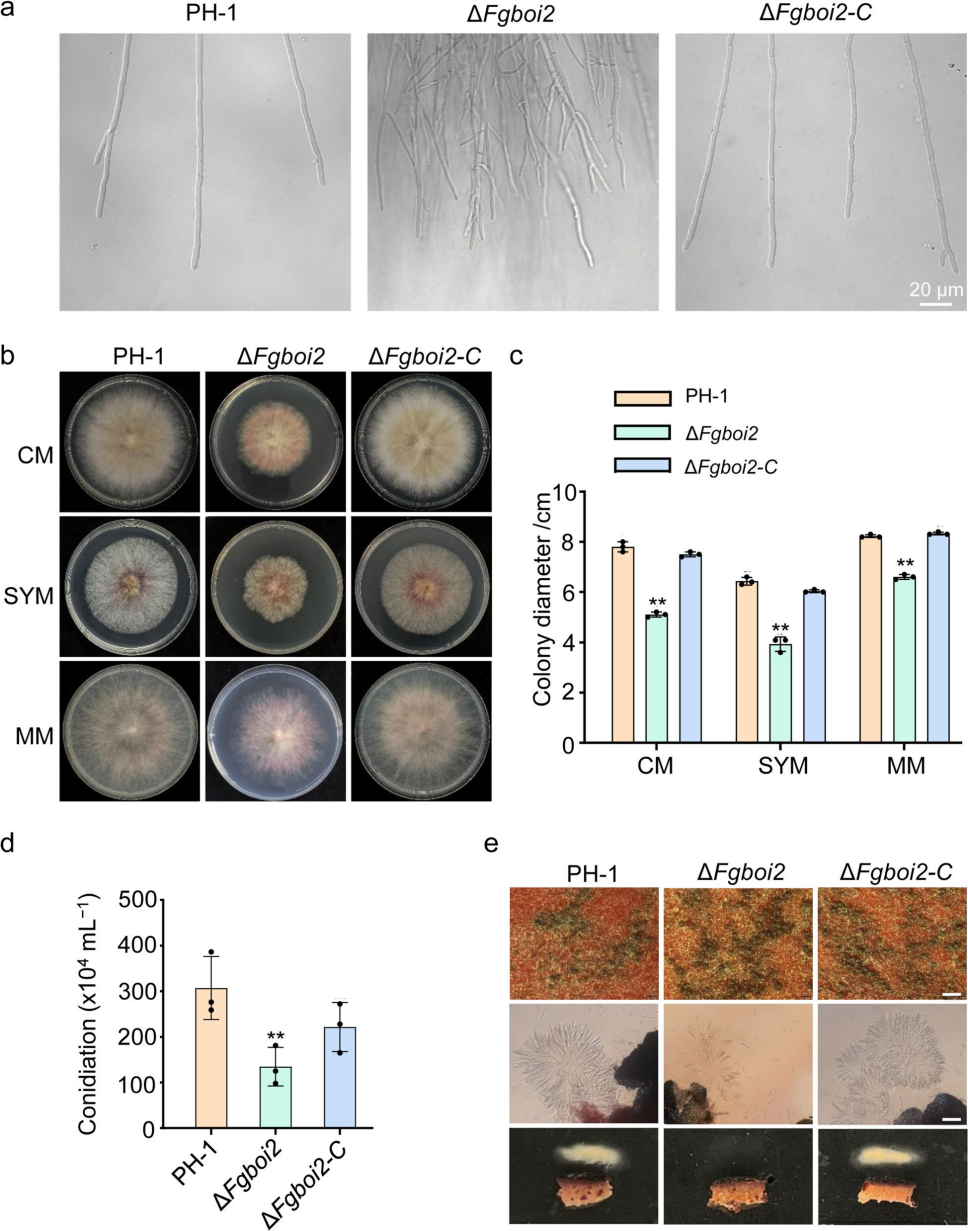
Role of FgBoi2 in vegetative growth, asexual and sexual reproduction of *Fusarium graminearum*. **a** Hyphal tip growth and branching patterns of PH-1, *FgBOI2* deletion mutant (*∆Fgboi2*) and complemented strain (*∆Fgboi2-C*) on starch-yeast medium (SYM) plates. **b**-**c** The colony morphologies and diameters of PH-1, *∆Fgboi2* and *∆Fgboi2-C* on complete medium (CM), SYM and minimal medium (MM) plates for 3 days. Error bars represent mean ± SD from three replicates. Two-tailed Student’s *t*-test was used for paired comparison of the colony diameter between PH-1 and *∆Fgboi2* mutant (**, *P* < 0.01). **d** FgBoi2 is required for conidiation in *F*. *graminearum*. Error bars represent mean ± SD from three replicates. Two-tailed Student’s *t*-test was used for paired comparison of conidiation between PH-1 and *∆Fgboi2* mutant (**, *P* < 0.01). **e** FgBoi2 is important for ascospore release in *F*. *graminearum*. Top bar = 500 μm, bottom bar = 50 μm

To determine the role of FgBoi2 in hyphal tip growth and branching patterns of *F. graminearum*, the wild type PH-1, *FgBOI2* gene deletion mutant (*∆Fgboi2*) and complemented strain (*∆Fgboi2-C*) were inoculated on starch-yeast medium (SYM) plates and incubated for two days, after which their colony morphology was observed under a confocal microscope. The assay indicated numerous hyphal branching in the *∆Fgboi2* mutant which was not observed in PH-1 and *∆Fgboi2-C* strains (Fig. 3a). Also, the hyphal tip (an actively growing zone) of the *∆Fgboi2* mutant was thinner than that of PH-1 (Fig. 3a). Furthermore, Fig. 3b-c shows that the radial growth of the *∆Fgboi2* mutant was significantly reduced on CM, SYM and MM plates compared to those of the wild type PH-1 and the complemented strain *∆Fgboi2-C*. These results suggest that FgBoi2 is required for the polarized vegetative growth of *F. graminearum*.

### FgBoi2 plays a critical role in both asexual and sexual development of *F. graminearum*

Asexual and sexual reproduction are two important life cycle stages of *F. graminearum* and remain the major stages at which the fungus infects flowering wheat heads (Francl et al. 1999). To determine the role of FgBoi2 in the asexual development of *F. graminearum*, PH-1, *∆Fgboi2* and *∆Fgboi2*-C strains were inoculated in carboxymethylcellulose (CMC) media and incubated for 3 days at 28℃ for conidia production. As shown in Fig. 3d, deletion of *FgBOI2* caused significant reduction in conidiation compared to PH-1 and *∆Fgboi2*-C strains. However, we found that FgBoi2 is not required for perithecia and ascospore formation, but ascospore release process was significantly affected in the mutant (Fig. 3e). Taken together, these results indicate that FgBoi2 is important for the normal asexual and sexual development of* F. graminearum*.

### FgBoi2 is important for pathogenicity and DON production in *F. graminearum*

**Fig. 4:**
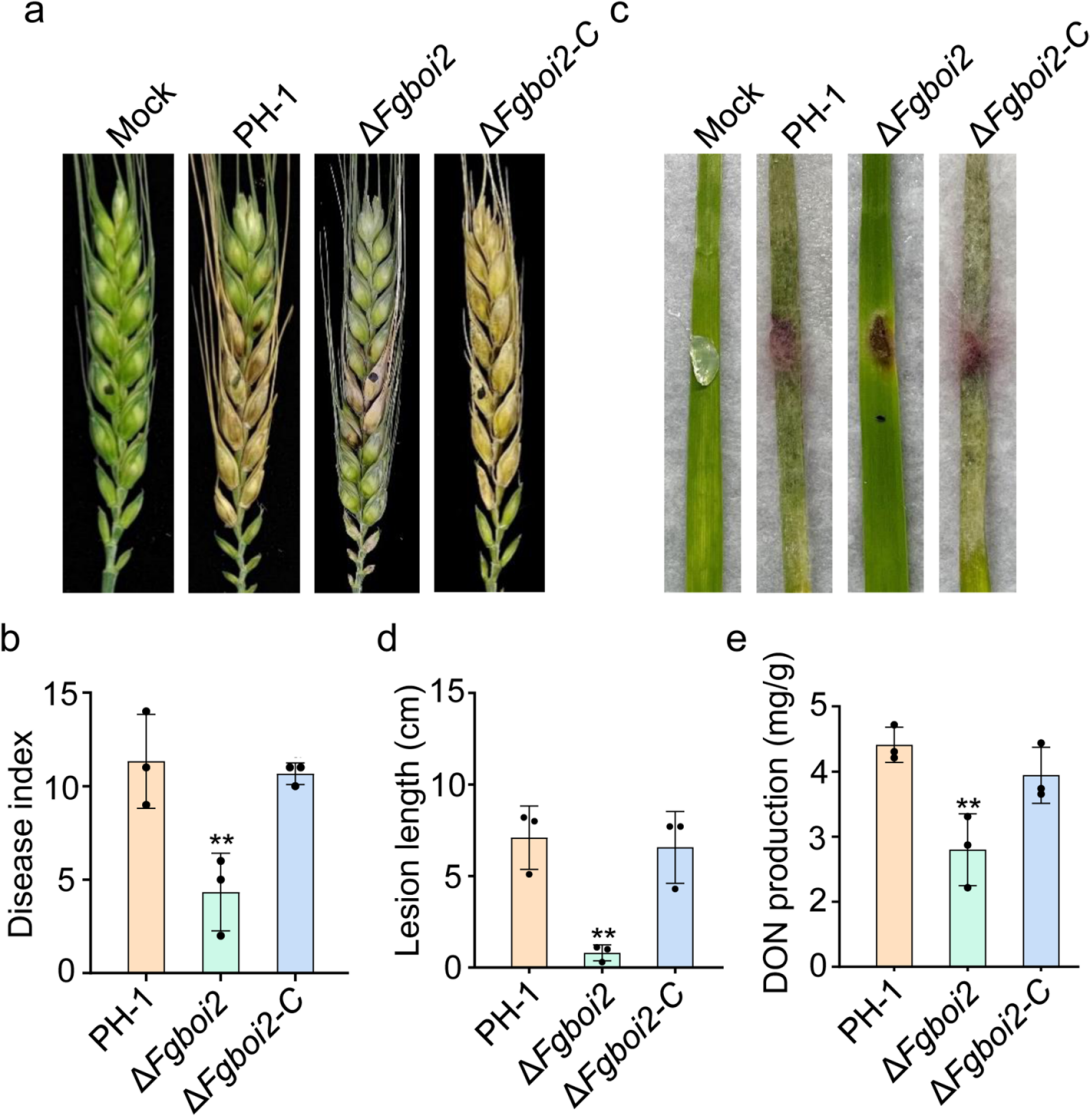
FgBoi2 regulates the pathogenicity and DON production of *Fusarium graminearum*. **a** The pathogenicity of the wild type PH-1 and *∆Fgboi2* mutant on wheat heads. Images of infected wheat heads were taken 14 days after inoculation with fresh mycelia from each strain. **b** Disease index rated based on the number of symptomatic spikelets. Error bars represent mean ± SD from three replicates. Two-tailed Student’s *t*-test was used for paired comparison of disease index between PH-1 and *∆Fgboi2* mutant (**, *P* < 0.01). **c**-**d** Pathogenicity and lesion length of PH-1 and *∆Fgboi2* mutant inoculated on wheat seedling leaves for 7 days. **e** Deoxynivalenol (DON) production of the wild type PH-1, *∆Fgboi2* and *∆Fgboi2-C* in liquid trichothecene biosynthesis-inducing (TBI) medium after 7 days of incubation. Error bars represent mean ± SD from three replicates. Two-tailed Student’s *t*-test was used for paired comparison of DON production between PH-1 and *∆Fgboi2* mutant (**, *P* < 0.01)

To investigate the role of FgBoi2 in the pathogenicity of *F. graminearum*, the wild type PH-1, *∆Fgboi2* mutant and *∆Fgboi2-C* strains were inoculated on flowering wheat heads under moist condition for 14 days. After this period, we observed that the PH-1, *∆Fgboi2* and *∆Fgboi2-C* strains were able to spread from the inoculated spikelet to the neighboring spikelets and induced typical Fusarium head blight symptoms (Fig. 4a). However, the average disease index (diseased spikelets per head) of *∆Fgboi2* mutant was significantly lower than those of PH-1 and *∆Fgboi2-C* strains (Fig. 4b), suggesting the important role of FgBoi2 in the pathogenicity of *F. graminearum*. Consistently, the average length of disease lesion inflicted by *∆Fgboi2* mutant on the leaves of wheat seedlings was also significantly reduced compared to PH-1 and *∆Fgboi2-C* strains (Fig. 4c-d). Taken together, these results suggest that FgBoi2 contributes to *F. graminearum* pathogenicity to host plants.

DON is a key virulence factor produced by *F. graminearum*, which facilitates the fungal spread during plant infection (Proctor et al. 1995; Chen et al. 2019). To investigate the role of FgBoi2 in DON production of *F. graminearum*, the wild type PH-1, *∆Fgboi2* mutant and *∆Fgboi2-C* strains were inoculated in liquid TBI media for 7 days at 28℃ in the dark. As shown in Fig. 4e, the level of DON produced by the *∆Fgboi2* mutant was significantly reduced in comparison to those produced by PH-1 and *∆Fgboi2-C* strains, indicating that FgBoi2 is required for normal DON production in *F. graminearum*.

### The PH domain of FgBoi2 contributes to its correct localization and biological function in *F. graminearum*

**Fig. 5:**
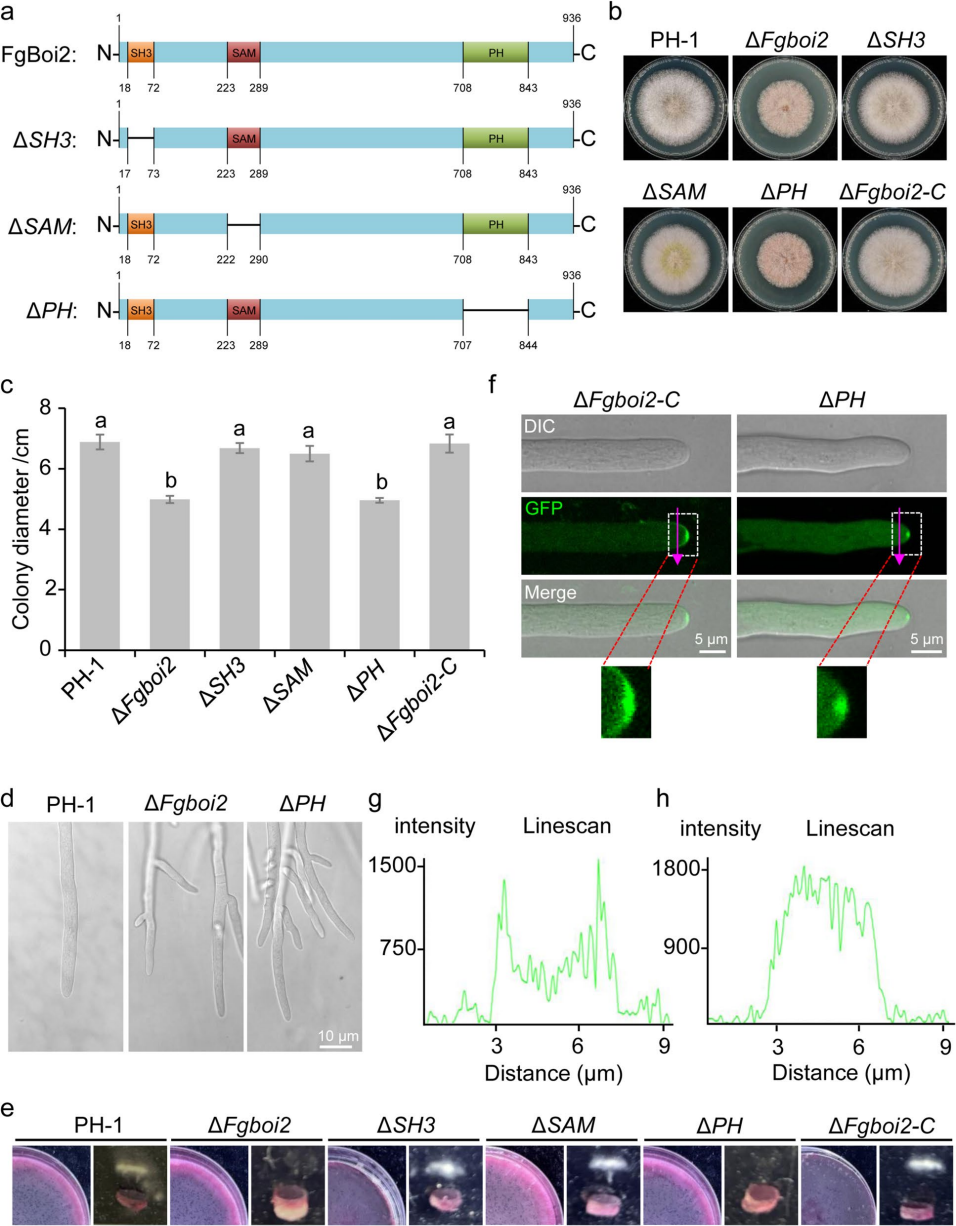
Functional characterization of the SH3, SAM, and PH domains of FgBoi2. **a** Schematic diagram of FgBoi2-full length, SH3, SAM and PH domain deletion mutants. **b** The colony morphologies of PH-1, Δ*Fgboi2*, Fgboi2^Δ*SH3*^ (Δ*SH3*), Fgboi2^Δ*SAM*^ (Δ*SAM*) and Fgboi2^Δ*PH*^ (Δ*PH*) on CM after 3 days. **c** The colony diameters of the indicated strains on CM after 3 days. Error bars represent mean ± SD from three replicates. Bars with same letters are not significantly different at *P* < 0.05. **d** Hyphal tip growth and branching patterns of PH-1, Δ*Fgboi2* and PH domain deletion mutant (Δ*PH*) on starch-yeast medium (SYM) plates. **e** PH domain of FgBoi2 is required for the ascospore release. **f** PH domain is essential for correct localization of FgBoi2-GFP. **g** Line scans showing the localization of FgBoi2-GFP in the plasma membrane (purple arrows) of Fig. 4d. **h** Line scans showing the localization of Fgboi2^Δ*PH*^-GFP in the plasma membrane (purple arrows) of Fig. 4d

Bioinformatic analysis revealed that FgBoi2 contains three conserved domains: SH3, SAM and PH domains (Fig. 5a). To gain an insight into the functional mechanism of FgBoi2, we decided to investigate which of these domains is/are important for FgBoi2 function. To achieve this, we generated pFgBoi2^*∆SH3*^ (lacking the SH3 domain, 18–72 aa), pFgBoi2^*∆SAM*^ (lacking the SAM domain, 223–289 aa) and pFgBoi2^∆*PH*^ (lacking the PH domain, 708–843 aa) constructs (Fig. 5a) and transformed them into the protoplasts of *∆Fgboi2* mutant, respectively, to obtain PH, SAM and SH3 domain deletion mutants of FgBoi2. We then analyzed the phenotypes of each of these mutants and found that the PH domain of FgBoi2 is critically important for the vegetative growth of *F. graminearum*, while the SAM and SH3 domains only play a minor role (Fig. 5b-c). Moreover, like the deletion of *FgBOI2*, the absence of the PH domain resulted in increased hyphal branching, with the formation of thinner hyphae compared to PH-1 (Fig. 5d). In addition, the sexual development experiment shows that the PH domain of FgBoi2 is required for ascospore release while SAM and SH3 domains of FgBoi2 are not required for this process (Fig. 5e). These results suggest that the PH domain of FgBoi2 is crucial for its biological functions in *F. graminearum*. Furthermore, subcellular localization experiment shows that the PH domain is required for the hyphae tip localization of FgBoi2-GFP (Fig. 5f), as deletion of the PH domain caused mis-localization of Fgbio2^*∆PH*^-GFP to cytoplasmic puncta (Fig. 5f). Consistently, line scans further confirm the mis-localization of Fgbio2^*∆PH*^-GFP fluorescence signal from the plasma membrane (Fig. 5g-h), suggesting that the PH domain is required for FgBoi2 anchorage to the plasma membrane of hyphal tips in *F. graminearum*. Taken together, these results indicate that the PH domain of FgBoi2 is essential for its function and correct localization in *F. graminearum*.

### FgBoi2 is involved in abiotic stress responses in *F. graminearum*

**Fig. 6:**
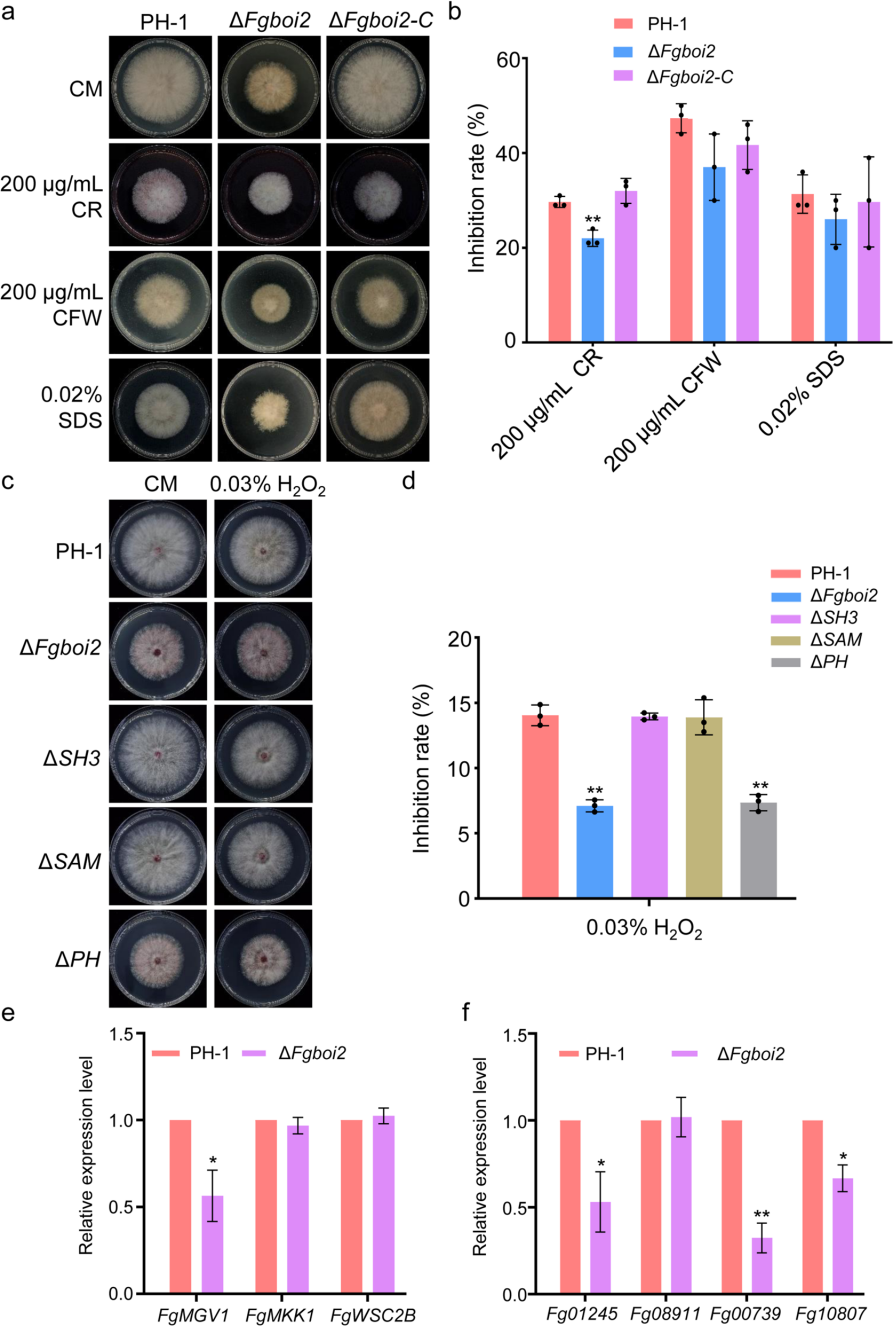
FgBoi2 negatively regulates responses to various environmental stress factors in *Fusarium graminearum*. **a** The colony morphology of PH-1, Δ*Fgboi2* and Δ*Fgboi2*-*C* strains grown on CM plates supplemented with Congo red (CR, 200 μg/mL), calcofluor white (CFW, 200 μg/mL), and SDS (0.02%). **b** Mycelial inhibition rate of PH-1, Δ*Fgboi2* and Δ*Fgboi2*-*C* strains grown on CM plates supplemented with CR, CFW, SDS. Error bars represent mean ± SD from three replicates. Two-tailed Student’s *t*-test was used for paired comparison of inhibition rate between PH-1 and *∆Fgboi2* mutant (**, *P* < 0.01). **c**-**d** The colony morphology and mycelial inhibition rate of PH-1, Δ*Fgboi2*, Fgboi2^Δ*SH3*^ (Δ*SH3*), Fgboi2^Δ*SAM*^ (Δ*SAM*) and Fgboi2^Δ*PH*^ (Δ*PH*) on CM plates supplemented with the oxidative stress agent H_2_O_2_ (0.03%). Two-tailed Student’s *t*-test was used for paired comparison of inhibition rate between PH-1 and indicated mutant (**, *P* < 0.01). **e** The relative expression levels of CWI genes (*FgMGV1*, *FgMKK1*, *FgWSC2B*) in the Δ*Fgboi2* mutant and wild type PH-1. Two-tailed Student’s *t*-test was used for paired comparison of relative expression levels between PH-1 and *∆Fgboi2* mutant (*, *P* < 0.05). **f** The relative expression levels of catalase, peroxidase, NADPH oxidase superfamily related genes (FGSG_01245, FGSG_08911, FGSG_00739, FGSG_10807) in the Δ*Fgboi2* mutant and wild type PH-1. Two-tailed Student’s *t*-test was used for paired comparison of relative expression levels between PH-1 and *∆Fgboi2* mutant (*, *P* < 0.05; **, *P* < 0.01)

During the process of plant infection, the sensitivity of *F. graminearum* to environmental stresses is very important (Leplat et al. 2013). To determine the role of FgBoi2 in sensitivity to environmental stress, we examined the response of *∆Fgboi2* to various environmental stress factors, including the cell wall damaging agents Congo Red (CR, 200 μg/mL) and calcofluor white (CFW, 200 μg/mL), the cell membrane damaging agent (SDS, 0.02%) and the oxidative stress agent H_2_O_2_ (0.03%). As shown in Fig. 6a-d, the sensitivity of *∆Fgboi2* to CR and H_2_O_2_ stresses was significantly reduced compared to the wild type PH-1 and complemented strain *∆Fgboi2-C*. In addition, the sensitivity of *∆Fgboi2* to CFW and SDS stresses was slightly decreased compared to PH-1 and *∆Fgboi2-C* (Fig. 6a-b). Furthermore, our result showed that the PH domain of FgBoi2 plays a critical role in oxidative stress tolerance (Fig. 6c-d). These results suggest that FgBoi2 is involved in the regulation of sensitivity to various environmental stresses.

To further investigate the role of FgBoi2 in negatively regulating the cell wall integrity (CWI) and oxidative stress pathways, we assayed the relative expression levels of CWI genes (*FgMGV1*-*FGSG_10313*, *FgMKK1-FGSG_07295*, *FgWSC2B-FGSG_10787*) (Hou et al. 2002; Yun et al. 2014; Xu et al. 2019) and catalase, peroxidase, NADPH oxidase superfamily related genes (FGSG_01245, FGSG_08911, FGSG_00739, FGSG_10807) (Lee et al. 2018; Luo et al. 2025) in the Δ*Fgboi2* mutant vs wild type PH-1. As shown in Fig. 6e-f, we observed that the relative expression levels of *FgMGV1*, FGSG_01245, FGSG_00739, FGSG_10807 were significantly decreased in the Δ*Fgboi2* mutant compared to the wild type PH-1. Taken together, these results indicate that FgBoi2 negatively regulates cell wall integrity and oxidative stress response by modulating the expression of CWI and catalase, peroxidase, NADPH oxidase superfamily related genes.

### Deletion of *FgPEA2* or *FgBOI2* reduces sensitivity to fungicides

**Fig. 7:**
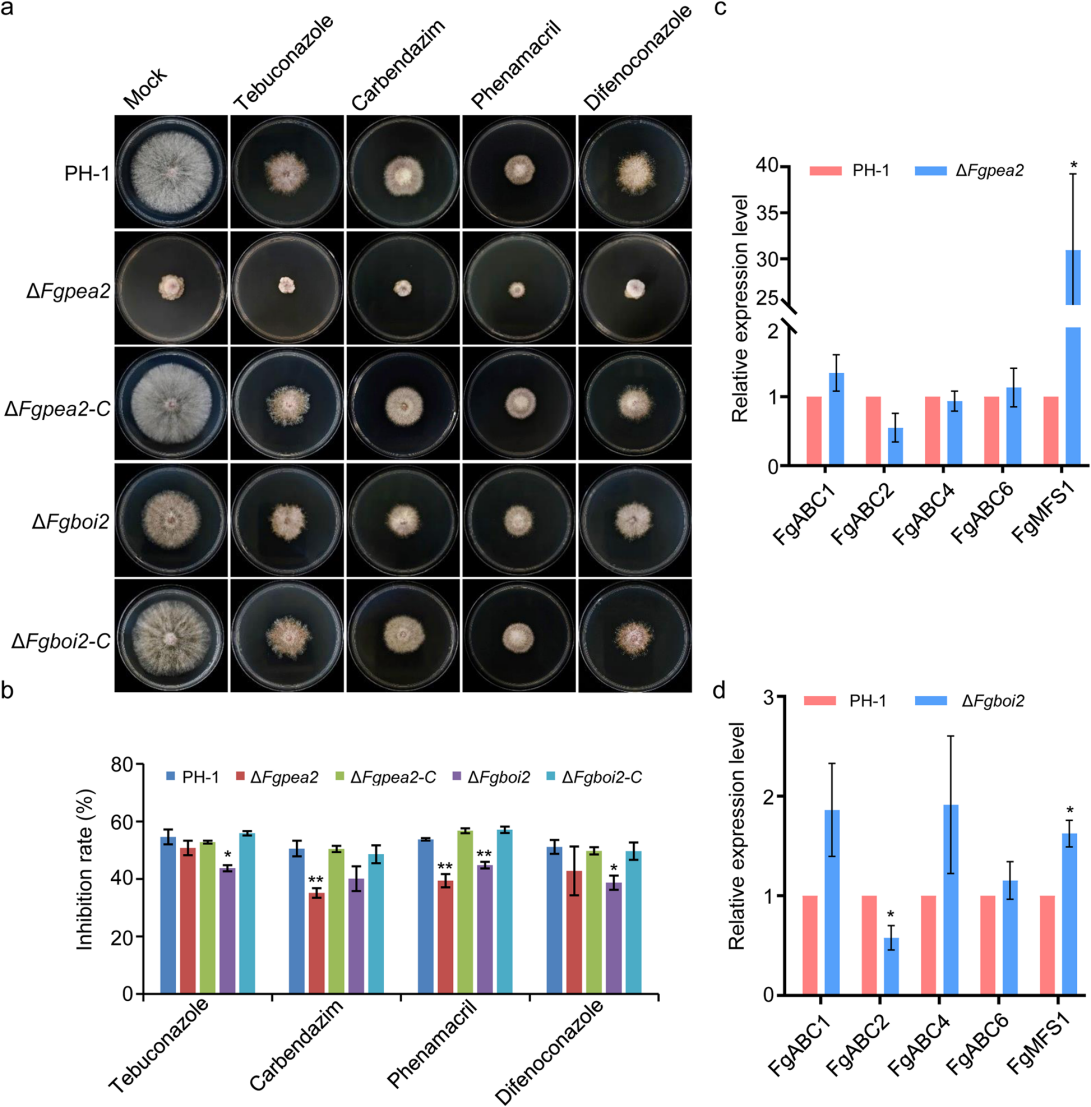
FgPea2 and FgBoi2 regulate the sensitivity of *Fusarium graminearum* to tebuconazole, carbendazim, phenamacril and difenoconazole. **a** Δ*Fgboi2* and Δ*Fgpea2* mutants have reduced sensitivity to 0.6 μg/mL tebuconazole, 0.8 μg/mL carbendazim, 0.5 μg/mL phenamacril and 1.5 μg/mL difenoconazole. **b** Statistical analysis of the growth inhibition rate of all strains under tebuconazole, carbendazim, phenamacril and difenoconazole stress. Error bars represent mean ± SD from three replicates. Two-tailed Student’s *t*-test was used for paired comparison of inhibition rate between PH-1 and *∆Fgboi2* or *∆Fgpea2* mutant (*, *P* < 0.05; **, *P* < 0.01). **c** The relative expression levels of *FgABC1* (FGSG_04580), *FgABC2* (FGSG_17046), *FgABC4* (FGSG_17058), *FgABC6* (FGSG_11028) and *FgMFS1* (FGSG_07802) in the Δ*Fgpea2* mutant and wild type PH-1. Two-tailed Student’s *t*-test was used for paired comparison of relative expression levels between PH-1 and *∆Fgpea2* mutant (*, *P* < 0.05). **d** The relative expression levels of *FgABC1* (FGSG_04580), *FgABC2* (FGSG_17046), *FgABC4* (FGSG_17058), *FgABC6* (FGSG_11028) and *FgMFS1* (FGSG_07802) in the Δ*Fgboi2* mutant and wild type PH-1. Two-tailed Student’s *t*-test was used for paired comparison of relative expression levels between PH-1 and *∆Fgboi2* mutant (*, *P* < 0.05)

Fungicides are currently the main method to control FHB (Mesterházy et al. 2011). To determine the effect of deleting *FgBOI2* gene on the susceptibility of *F. graminearum* to fungicides, we selected some common fungicides used for controlling FHB, including tebuconazole, carbendazim, phenamacril and difenoconazole. The wild type PH-1, *∆Fgboi2* and complemented strain *∆Fgboi2-C* were inoculated on CM plates containing 0.6 μg/mL tebuconazole, 0.8 μg/mL carbendazim, 0.5 μg/mL phenamacril and 1.5 μg/mL difenoconazole, respectively. As shown in Fig. 7a-b, deletion of *FgBOI2* reduces the sensitivity to the fungicides tebuconazole, carbendazim, phenamacril and difenoconazole compared to the wild type and complemented strain, suggesting that FgBoi2 is involved in fungicide resistance in *F. graminearum*. To find out whether FgPea2 is involved in fungicide resistance, PH-1, *∆Fgpea2* and *∆Fgpea2-C* strains were similarly inoculated on CM agar plates containing aforementioned fungicides. The result shows that FgPea2 is also involved in fungicide resistance. Furthermore, we showed that the EC_50_ values of wild type PH-1 to tebuconazole, carbendazim, phenamacril and difenoconazole are 0.45, 0.76, 0.51 and 0.99 ug/mL, respectively. The EC_50_ values of the *∆Fgboi2* against these fungicides are 0.54, 0.84, 0.55, 1.45 ug/mL, respectively. The EC_50_ values of *∆Fgpea2* against these fungicides are 0.56, 0.96, 0.69 and 1.29 ug/mL, respectively. Taken together, these results suggest that *∆Fgboi2* and *∆Fgpea2* mutants have greater resistance to these fungicides than wild type PH-1 in *F. graminearum*..

To dissect the potential regulatory mechanisms of FgPea2 or FgBoi2 reduces sensitivity to multiple fungicides, we assayed some reported efflux transporters’ gene in the Δ*Fgboi2* and Δ*Fgpea2* mutants, for example: the ATP-binding cassette (ABC) transporter class genes *FgABC1* (FGSG_04580), *FgABC2* (FGSG_17046), *FgABC4* (FGSG_17058), *FgABC6* (FGSG_11028) (Abou Ammar et al. 2013; Gardiner et al. 2013; O’Mara et al. 2023) and the major facilitator superfamily (MFS) transporter gene *FgMFS1* (FGSG_07802) (O’Mara et al. 2023). As shown in Fig. 7c-d, the relative expression levels of *FgABC1*, *FgABC6* and *FgMFS1* genes were increased in the Δ*Fgboi2* and Δ*Fgpea2* mutants compared to the wild type PH-1. In addition, the relative expression level of *FgABC2* gene was decreased in the Δ*Fgboi2* and Δ*Fgpea2* mutants (Fig. 7c-d). Collectively, these results suggest that FgPea2 and FgBoi2 reduce sensitivity to multiple fungicides by regulating the transcriptional level of drug resistance ABC and MFS transporters.

## Discussion

Hyphal tip growth is a key feature of filamentous fungi and is critical for plant infection in *F. graminearum* (Lou et al. 2021; Zheng et al. 2021). However, the molecular mechanisms regulating cell polarity or tip growth are still poorly understood in *F. graminearum*. Our previous study has shown that the polarisome complex is required for polarized growth and plant infection in *F. graminearum*. We have shown that the FgMsb3–FgRab8 cascade is modulated by the polarisome complex, probably in a spatially distinct manner (Zheng et al. 2021). Furthermore, the exact spatiotemporal assembly and roles of the polarisome components in *F. graminearum* have been established. To further reveal the regulatory mechanism of the polarisome in *F. graminearum*, we performed a pull down assay of the polarisome core component FgPea2-GFP, followed by LC–MS/MS. In this study, the anillin-related protein Boi1/2 (FgBoi2) was identified from FgPea2-GFP pull down. However, the functions of Boi1/2 homologous proteins remain unknown in plant pathogenic fungi. Therefore, we systematically analyzed the functions of FgBoi2 and found that the polarized localization of FgBoi2 is regulated by FgPea2. Phenotypic identification showed that FgBoi2 is required for polarized growth, DON production and pathogenicity of *F. graminearum*. Interestingly, the PH domain present in FgBoi2 is essential for the polarized growth and plasma membrane localization of the protein. Furthermore, fungicide sensitivity assay showed that FgPea2 and FgBoi2 modulate susceptibility to fungicides tebuconazole, carbendazim, phenamacril and difenoconazole in *F. graminearum*.

Genetic analysis revealed that Boi1 and Boi2 are not only important for cell growth but also functionally redundant (Matsui et al. 1996). Boi1/2 are plasma membrane proteins that function in polarized growth and inhibit cytokinesis in response to chromosome bridges via the NoCut abscission checkpoint (Masgrau et al. 2017). Loss of either Boi1 or Boi2 has no effect on cell growth, but the simultaneous absence of both proteins is lethal (Bender et al. 1996; Matsui et al. 1996; Sulpizio et al. 2022). Moreover, overexpression of *BOI1* gene inhibits cell growth (Matsui et al. 1996). However, *F. graminearum* harbors only one Boi protein (FgBoi2), suggesting a divergence of Boi homologous proteins through evolution in fungi. Boi1 and Boi2 (Boi1/2) were first identified as interacting proteins of Bem1 (Matsui et al. 1996). A two-hybrid screening of Bem1 interactome identified Boi1 (Bem One Interactor 1) and its homolog Boi2 as binding targets of SH3-2 domain of Bem1 in yeast. In *Aspergillus nidulans*, BemA/1 was identified as a potential polarisome component (Leeder and Turner 2008). In *F. graminearum*, FgBem1 targets to septal pores and surface crescent at the apical hyphae and is necessary for normal nuclear division and pathogenicity (Lou et al. 2021) but dispensable for polarized secretion. Unlike Bem1, Boi1/2 interacts with Msb1, a protein that is required for the synthesis of 1,3-β-glucan in cell walls (Sekiya-Kawasaki et al. 2002) and localizes to sites of polarized growth during bud development and interacts with Cdc42 in the cell (Liao et al. 2013). In this study, FgBoi2 was identified as a new interacting protein of FgPea2, and FgPea2 regulates FgBoi2-mediated growth, development and virulence in *F. graminearum*. FgBoi2 interacts with FgPea2 and exhibits partial co-localization at the hyphal tips of *F. graminearum*. Deletion of *FgPEA2* disrupts the polarisome-like localization of FgBoi2 in hyphae tips. We speculate that FgPea2 may recognize and recruit FgBoi2 to the polarisome (SPK) region. In addition, based on the localization patterns of FgPea2 and FgBoi2 (Fig. 1c, e), we observed only partial co-localization between them. Notably, FgBoi2 localizes to apical plasma membrane (cortex) of the hyphae (Fig. 1c), suggesting distinct functional roles of FgPea2 and FgBoi2. For instance, the growth rate of ∆*Fgpea2* mutant was significantly slower than that of the ∆*Fgboi2* mutant.

Boi1 and Boi2 are functionally redundant in promoting the fusion of secretory vesicles with the plasma membrane at sites of polarized growth, and act as abscission inhibitors during cytokinesis in response to chromatin bridges (Masgrau et al. 2017). Cells lacking Boi1/2 accumulate secretory vesicles and are defective in bud growth (Masgrau et al. 2017). In this study, deletion of FgBoi2 did not significantly affect the polarized trafficking and exocytosis of the v-SNARE protein GFP-FgSnc1, suggesting that FgBoi2 is not involved in FgSnc1-mediated polarized trafficking and exocytosis in *F. graminearum*. Furthermore, BFA does not affect the polarized localization or trafficking of FgBoi2-GFP in *F. graminearum*. Boi1/2 recruit nucleation and elongation factors to form actin filaments at sites of exocytosis in yeast (Glomb et al. 2020). The proteins interact with Bud6 and Bni1 to induce the formation of a cortical actin structure that receives and aligns incoming vesicles before fusion with target membranes (Glomb et al. 2020). Disrupting the interaction of Boi1/2 and Bud6 impairs filament formation, reduces the directed movement of vesicles to hyphal tips and shortens the vesicles’ tethering time at the cortex (Glomb et al. 2020). However, in our study, we could not find FgBoi2 in FgBud6-GFP pull down data (Zheng et al. 2021), suggesting that FgBoi2 does not directly interact with FgBud6 in *F. graminearum*, indicating potentially distinct function from its yeast counterpart.

Domain structural analysis revealed that Boi2 contains SH3, SAM and PH domains. The SH3 domain of Boi2, which is dispensable for bud growth and targets Boi2 to the site of abscission, is necessary and sufficient for abscission inhibition (Masgrau et al. 2017). Boi1 binds to the plasma membrane of the bud through its PH domain (Hallett et al. 2002; Jia et al. 2020). In this study, the SH3 domain of FgBoi2 only contributes a little role to the function of FgBoi2 while the PH domain is crucial for hyphal polarized growth and plasma membrane localization of *F. graminearum*, indicating an essential role of the PH domain in anchoring FgBoi2 to the plasma membrane.

Environmental stress is an important factor that determines *F. graminearum* infection (Leplat et al. 2013; Sun et al. 2025). In this study, we found that FgBoi2 is involved in various abiotic stress responses as its absence in *∆Fgboi2* mutant improved the mutant’s resistance to CR, H_2_O_2_, CFW and SDS stress factors, suggesting a negative regulatory role of FgBoi2 in environmental stress response. In addition, the use of fungicides is the main method for controlling FHB in the field all over the world (Mesterházy et al. 2011; Tini et al. 2020). We showed that the absence of FgPea2 and FgBoi2 promotes the fungal resistance to tebuconazole, carbendazim, phenamacril and difenoconazole by regulating the transcriptional level of drug resistance ABC and MFS transporters, suggesting that disruption of hyphal tip growth decreased the sensitivity to these fungicides in *F. graminearum*. In the future, developing a fungicide that inhibits *F. graminearum* tip growth could serve as an effective strategy for controlling FHB.

## Conclusion

In summary, this study uncovers a novel regulatory mechanism of FgPea2 and FgBoi2 functions in *F. graminearum*, including regulation of hyphal tip growth, development, DON production, fungicide resistance and virulence.

## Materials and methods

### Strains and culture conditions

The *F. graminearum* wild type PH-1 was used as the background strain from which all mutant strains were generated. All the strains used in this study are listed in Table S1. All the strains were cultured on complete medium (CM, 10 g/L sucrose, 6 g/L casein hydrolysate, 6 g/L yeast extract, or 20 g/L agarose for solid medium), starch yeast medium (SYM, 2 g/L yeast extract, 10 g/L starch, 3 g/L sucrose, or 20 g/L agarose for solid medium), or minimal media (MM, 0.312 g/L MgSO_4_•7H_2_O, 0.52 g/L KCl, 1 g/L KH_2_PO_4_, 1 mL/L vitamin solution, 1 mL/L trace elements, 6 g/L NaNO_3_, 10 g/L glucose, or 20 g/L agarose for solid medium) at 28℃ for 3 days. Asexual and sexual (perithecia formation) reproduction were performed as previously reported (Zheng et al. 2015; Mao et al. 2024).

### *F. graminearum* transformation and generation of gene deletion mutants

*F. graminearum* protoplast preparation and transformations were carried out following standard protocols as previously reported (Hou et al. 2002). The split-marker approach (Catlett et al. 2003) was used to generate gene replacement construct for the *FgBOI2* gene. The primers used to amplify the flanking sequences for *FgBOI2* gene are listed in Table S2. Hygromycin-resistant transformants were screened by PCR with primer pairs *FgBOI2-*OF/*FgBOI2-*OR and *FgBOI2*-UA/H853 (Table S2). Subsequently, knockout candidates were further verified by Southern blot.

### Construction of pFgBoi2-GFP, pFgBoi2^∆*SH3*^-GFP, pFgBoi2^∆*SAM*^-GFP and pFgBoi2^∆*PH*^-GFP vectors and complementation

pFgBoi2-GFP fusion vector was constructed by amplifying the full-length sequence of the *FgBOI2* gene with primer pairs *FgBOI2*-CF and *FgBOI2*-CR (Table S2) from the WT genomic DNA. The sequence was then cloned into pKNTG vector using a One Step Cloning Kit (Vazyme Biotech Co. Ltd, Nanjing, China) and verified by sequencing. The pFgBoi2-GFP vector was then transformed into the protoplast of *∆Fgboi2* mutant, and a complemented strain was successfully generated which showed similar phenotypes to the PH-1. pFgBoi2^∆*SH3*^-GFP, pFgBoi2^∆*SAM*^-GFP and pFgBoi2^∆*PH*^-GFP vectors were constructed by amplifying their respectively sequences from the plasmid of pFgBoi2-GFP, using the primers listed in Table S2. Their sequences were then cloned into a pKNTG vector using a One Step Cloning Kit and verified by sequencing, respectively. The pFgBoi2^∆*SH3*^-GFP, pFgBoi2^∆*SAM*^-GFP and pFgBoi2^∆*PH*^-GFP vectors were transformed into the protoplasts of *∆Fgboi2* mutant, and SH3, SAM and PH domain deletion mutants of FgBoi2 were successfully generated.

### Co-immunoprecipitation (Co-IP) assays

pFgPea2-flag vector was constructed by amplifying the FgPea2 coding sequence together with its native promoter using the primers listed in Table S2. The amplicon was then cloned into pKNTG vector using a One Step Cloning Kit and verified by sequencing. For in vivo coimmunoprecipitation (Co-IP) assays, the construct pFgPea2-flag was transformed into the *∆Fgboi2* mutant with pCT74-sGFP (control), pFgBoi2-GFP, respectively. The resulting transformants were confirmed by western blot analysis using monoclonal anti-Flag (ab205606, Abcam, UK) and anti-GFP (M20004, Abmart, China). Total protein was extracted from the strain expressing the pair of fusion constructs and were further incubated with the anti-GFP agarose beads (SM038001, Smart-Lifesciences, China) at 4℃ for 4 h following the standard procedure (Mao et al. 2024). Finally, the total protein (Input) and the protein eluted from the agarose beads (IP) were subjected to western blot using anti-flag and anti-GFP antibodies, respectively.

### Plant infection and DON production assays

Infection assays of PH-1 and mutants on flowering wheat heads and wheat leaves were conducted as previously described (Miao et al. 2023; Chen et al. 2024). A mycelial agar block was inoculated on flowering wheat heads and wheat leaves and the infected plants were allowed to thrive for 14 and 7 days (Zhang et al. 2023; Luo et al. 2025), respectively, before being observed for lesion development. For DON production assays, all the strains were grown in liquid trichothecene biosynthesis induction (TBI) media at 28℃ for 7 days in the dark. The liquid was used for DON production assay while the mycelia were weighed and recorded after drying completely, as previously reported (Zheng et al. 2021). All experiments were repeated three times.

### Sensitivity of *F. graminearum* mycelial growth to multiple stresses

To determine the sensitivity of *∆Fgboi2* to various stresses, the colony diameters of the strain were measured after incubation on CM plates with or without the cell wall-damaging agents Congo Red (CR, 200 μg/mL) and calcofluor white (CFW, 200 μg/mL), the oxidative stress-inducing agent H_2_O_2_ (0.03%), the membrane stress agent SDS (0.02%) and fungicides (0.6 μg/mL tebuconazole, 0.8 μg/mL carbendazim, 0.5 μg/mL phenamacril, 1.5 μg/mL difenoconazole) for 3 days. The inhibition rate (%) was calculated by the following formula: (the averaged diameter of control − the averaged diameter in stresses)/(the averaged diameter of control − 5 mm of mycelial plug) × 100. All experiments were repeated three times.

### RNA extraction and qRT-PCR

For the qRT-PCR assay, wild type PH-1 and mutant strains were inoculated in CM for 2 days, fresh mycelia from each strain were frozen in liquid nitrogen. Total RNA extraction and subsequent synthesis of first-strand cDNA were performed as previously described (Yuan et al. 2022). The primer pairs used to amplify the selected genes in the qRT-PCR are listed in Table S2. All experiments were repeated three times.

### Live cell imaging of *F. graminearum*

Nikon Ci-S fluorescence microscope and Nikon CUS-W1 spinning-disk confocal microscope (Nikon, Tokyo, Japan) were used to observe the fluorescence signals of FgBoi2-GFP, FgPea2-GFP, FgPea2-mCherry and GFP-FgSnc1 fusion proteins. For hyphal tip and localization assays, a mycelial block from SYM agar containing the leading hyphae was excised, placed upside down on a coverslip, and observed directly. GFP and mCherry excitations were set at 488 and 561 nm, respectively.

### Accession numbers

*F. graminearum* (FgBoi2-XP_011318940.1),

*Fusarium oxysporum* (FoBoi2-SCO77667.1),

*Fusarium verticillioides* (FvBoi2-XP_018746470.1),

*Verticillium longisporum* (VlBoi2-KAG7148985.1),

*Neurospora crassa* (NcBoi2-XP_960881.3),

*Pyricularia oryzae* (PoBoi2-ELQ39503.1),

*Parastagonospora nodorum* (PnBoi2-KAH3911521.1),

*Saccharomyces cerevisiae* (ScBoi2-KZV11882.1),

*Schizosaccharomyces pombe* (SpBoi2-NP_596828.1).

## Supplementary Information

Supplementary Material 1: Fig. S1. Identification of FgBoi2 in *Fusarium graminearum.* a. Phylogenetic analysis of Boi2 in different organisms. The alignment of the retrieved protein sequences was conducted using MEGA 11.0 and phylogenetic tree was constructed by neighbor-joining method. b. Split-marker approach was used to delete the *FgBOI2* gene in *F. graminearum*. c. Southern blot confirmation of *FgBOI2* gene deletion in *F. graminearum* wild type PH-1 strain. *Xba*I (X) was used to digest the genomic DNA from the indicated strains which showed a 5.3 kb band in PH-1 and a 3.6 kb band in the *FgBOI2* gene deletion mutants. Fig. S2. The relative expression level of *FgBOI2* gene in the ∆*Fgpea2* mutant of *Fusarium graminearum.* Two-tailed Student’s *t*-test was used for paired comparison of the expression level of *FgBOI2* gene between PH-1 and *∆Fgpea2* mutant (*, *P* < 0.05). Table S1. Wild type (PH-1) and mutant strains used in this study. Table S2. PCR primers used in this study.

## Data Availability

All data generated or analyzed during this study are included in the published article and its supplementary information files.

## Abbreviations

DON: Deoxynivalenol
CM: Complete media
SYM: Starch yeast medium
MM: Minimal media
CR: Congo Red
CFW: Calcofluor white
SDS: Sodium dodecyl sulfate
CWI: Cell wall integrity
BFA: Brefeldin A
GFP: Green fluorescent protein
TBI: Trichothecene biosynthesis induction
