# Supplementary material for: Polarisome core component FgPea2 regulates FgBoi2-mediated polarized growth, pathogenicity and environmental stress in Fusarium graminearum
Source: Stress Biol. 2026 Apr 7;6(1):26. doi: 10.1007/s44154-026-00300-w (PMC13057054; doi:10.1007/s44154-026-00300-w)
Supplement: Supplementary file 1 — Supplementary Material 1: Fig. S1. Identification of FgBoi2 in Fusarium graminearum. a. Phylogenetic analysis of Boi2 in different organisms. The alignment of the retrieved protein sequences was conducted using MEGA 11.0 and phylogenetic tree was constructed by neighbor-joining method. b. Split-marker approach was used to delete the FgBOI2 gene in F. graminearum. c. Southern blot confirmation of FgBOI2 gene deletion in F. graminearum wild type PH-1 strain. XbaI (X) was used to digest the genomic DNA from the indicated strains which showed a 5.3 kb band in PH-1 and a 3.6 kb band in the FgBOI2 gene deletion mutants. Fig. S2. The relative expression level of FgBOI2 gene in the ∆Fgpea2 mutant of Fusarium graminearum. Two-tailed Student’s t-test was used for paired comparison of the expression level of FgBOI2 gene between PH-1 and ∆Fgpea2 mutant (*, P < 0.05). Table S1. Wild type (PH-1) and mutant strains used in this study. Table S2. PCR primers used in this study. [file 44154_2026_300_MOESM1_ESM.docx]

**Supplementary Information**

**
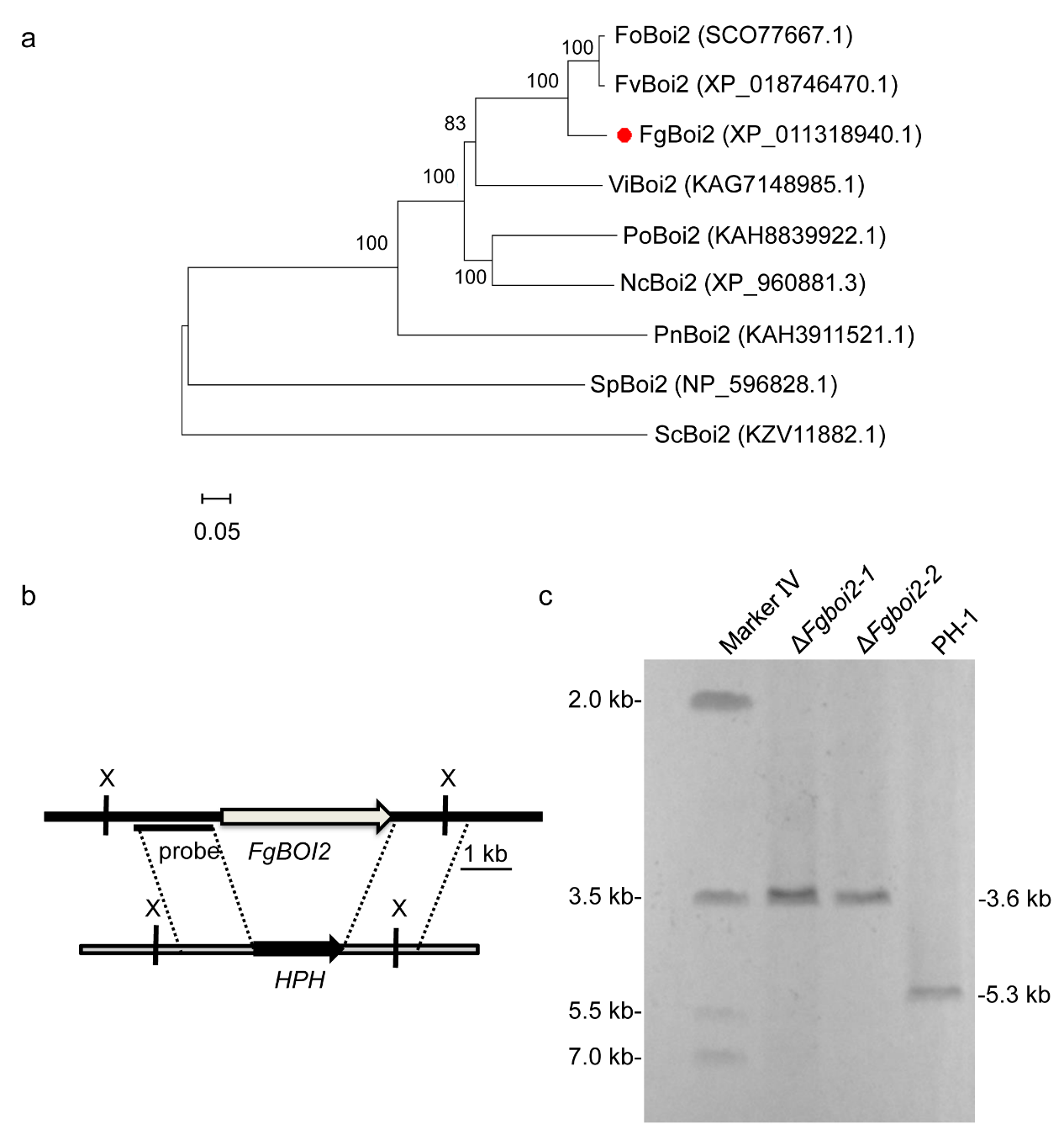
**

**Fig. S1 Identification of FgBoi2 in *Fusarium graminearum***

**a**. Phylogenetic analysis of Boi2 in different organisms. The alignment of the retrieved protein sequences was conducted using MEGA 11.0 and phylogenetic tree was constructed by neighbor-joining method.

**b**. Split-marker approach was used to delete the *FgBOI2* gene in *F. graminearum*.

**c**. Southern blot confirmation of *FgBOI2* gene deletion in *F. graminearum* wild type PH-1 strain. *Xba*I (X) was used to digest the genomic DNA from the indicated strains which showed a 5.3 kb band in PH-1 and a 3.6 kb band in the *FgBOI2* gene deletion mutants.

**
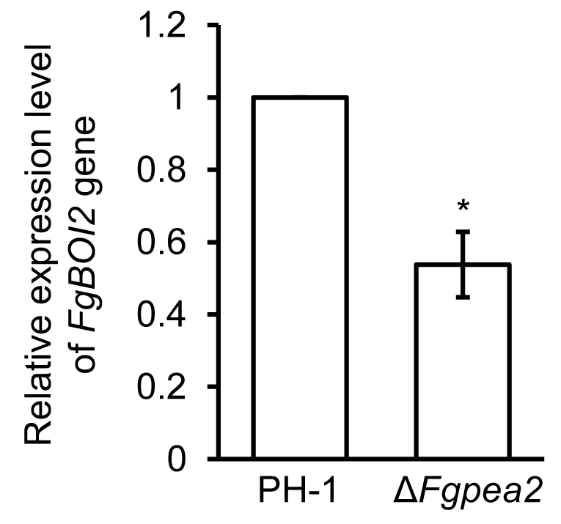
**

**Fig. S2 The relative expression level of *FgBOI2* gene in the ∆*Fgpea2* mutant of *Fusarium graminearum.***

Two-tailed Student’s *t*-test was used for paired comparison of the expression level of *FgBOI2* gene between PH-1 and *∆Fgpea2* mutant (*, *P* < 0.05).

**Table S1** Wild type (PH-1) and mutant strains used in this study.

| **Strain** | **Genotype description** | **Reference** |
| --- | --- | --- |
| PH-1 | Wild type | [^1^](#_ENREF_1) |
| Δ*Fgboi2* | FGSG_10016 deletion mutant in PH-1 | This study |
| Δ*Fgboi2-C* | Δ*Fgboi2* strain expressing the pKNTG-FgBoi2 construct | This study |
| *Fgboi2*^Δ^*^SH3^* | Δ*Fgboi2* strain expressing the pKNTG-FgBoi2^Δ^*^SH3^* construct | This study |
| *Fgboi2*^Δ^*^SAM^* | Δ*Fgboi2* strain expressing the pKNTG-FgBoi2^Δ^*^SAM^* construct | This study |
| *Fgboi2*^Δ^*^PH^* | Δ*Fgboi2* strain expressing the pKNTG-FgBoi2^Δ^*^PH^* construct | This study |
| PH-1+GFP-FgSnc1 | PH-1 strain expressing the GFP-FgSnc1 construct | [^2^](#_ENREF_2) |
| Δ*Fgboi2*+GFP-FgSnc1 | Δ*Fgboi2* strain expressing the GFP-FgSnc1 construct | This study |
| Δ*Fgpea2* | FGSG_05208 deletion mutant from PH-1 | [^3^](#_ENREF_3) |
| GFP+FgPea2-Flag | GFP and FgPea2-Flag transformant of PH-1 | This study |
| FgBoi2-GFP +FgPea2-Flag | FgBoi2-GFP and FgPea2-Flag transformant of PH-1 | This study |
| Δ*Fgpea2*+FgBoi2-GFP | FgBoi2-GFP transformant of Δ*Fgpea2* | This study |
| Δ*Fgboi2*+FgPea2-GFP | FgPea2-GFP transformant of Δ*Fgboi2* | This study |

**Table S2** PCR primers used in this study.

| **Primer** | **Sequence (5΄→3΄)** | **Application** |
| --- | --- | --- |
| *FgBOI2*-AF | TAGGCTGTGGGCAGAGGCA | *FgBOI2* deletion |
| *FgBOI2*-AR | TTGACCTCCACTAGCTCCAGCCAAGCCGCGGGTTGTGAAGAATGGTAT |  |
| *FgBOI2*-BF | GAATAGAGTAGATGCCGACCGCGGGTTGGGGCGTTTCTGGTCATTC |  |
| *FgBOI2*-BR | CAAGGCGTTCTGGAGGGTC |  |
| *FgBOI2*-OF | GACCCTTGGCAACGCTTAT |  |
| *FgBOI2*-OR | GGACTCGGTGGAATGAAAT |  |
| *FgBOI2*-UA | CTGGCACCATACGCACCTC |  |
| H853 | GACAGACGTCGCGGTGAGTT |  |
| YG/F | GATGTAGGAGGGCGTGGATATGTCCT |  |
| HY/R | GTATTGACCGATTCCTTGCGGTCCGAA |  |
| HYG/F | GGCTTGGCTGGAGCTAGTGGAGGTCAA |  |
| HYG/R | AACCCGCGGTCGGCATCTACTCTATTC |  |
| *FgBOI2*-CF | AGGGAACAAAAGCTGGGTACCTCCAATCCCAACATCCCT | complementation |
| *FgBOI2*-CR | GCCGCCGCCGCCGCCAAGCTTAGACGCGTGAGGAGCCCCT |  |
| SH3-R | GTCGCCAACTTCAGGCCTCG |  |
| SH3-F | CGAGGCCTGAAGTTGGCGACACCAGACTGGCCCCTAGAAA | Domain deletion |
| SAM-R | GACTTCTTCTCGTGTATGGA |  |
| SAM-F | TCCATACACGAGAAGAAGTCGATGAGGTTAACAACCAGGG |  |
| Boi2-PH-R | GGCATCTTTGCTTGCCTCCT |  |
| Boi2-PH-F | AGGAGGCAAGCAAAGATGCCATTGATCGAGACGATTCCAA |  |
| FGSG_04580QF | GGTCTCATGTGGCTGCTCAT | qPCR |
| FGSG_04580QR | ACCAGTCGACAGAATAGCCG |  |
| Fgβ-Tubulin QF | TCTGACTTCAGGAATGGTCGTTAC |  |
| Fgβ-Tubulin QR | AGCGGTCTGGATGTTGTTGG |  |
| FGSG_07802QF | TACTGGTCGATGGGTGGGAT |  |
| FGSG_07802QR | CTGAAATGTCGGATGCTGCG |  |
| FGSG_17046QF | GGAAGACTCTGGAAGCCACC |  |
| FGSG_17046QR | TCCGTGATTCGTGCCATCTC |  |
| FGSG_17058QF | ACGGAAGCTCCATCAAAGCA |  |
| FGSG_17058QR | TCCGCCCTGTTTCTTCTCAC |  |
| FGSG_11028QF | ATACGACGACGAGTGGCTTC |  |
| FGSG_11028QR | GGAGCGTTGTCCTTGTGAGA |  |
| FGSG_10313QF | CAGGAATACGTCCGCAACCT |  |
| FGSG_10313QR | GCATCTGACCAACGTCCTCA |  |
| FGSG_07295QF | AACCGGAGAGAAAGTGCTGG |  |
| FGSG_07295QR | CGACTGGCCTGTAATACGCT |  |
| FGSG_10787QF | TGGACCCGACAATTACGTGG |  |
| FGSG_10787QR | CTTGTAGCTGTGTCGTCGGT |  |
| FGSG_01245QF | GGATCTCTGGATCCTTGCCG |  |
| FGSG_01245QR | GAAACCCATACGGCCGAAGA |  |
| FGSG_08911QF | CCCGTCGGGATCATCAACAA |  |
| FGSG_08911QR | CAACGCGATCTCAGCACAAG |  |
| FGSG_00739QF | AACATCTGGCATCTCCGCAA |  |
| FGSG_00739QR | ATACGTGCTGCTTCGTTGGA |  |
| FGSG_10807QF | CAGCTATCCGTCGTGTCCTC |  |
| FGSG_10807QR | TCGAAGTCACGGCAAATCCA |  |
| FgBoi2-QF | CTGTCGAGGGCTGTGAACTT |  |
| FgBoi2-QR | AACGCATCTGCTTGGCTTTG |  |

**REFERENCES**

1. Cuomo, C. A.; Gueldener, U.; Xu, J. R.; Trail, F.; Turgeon, B. G.; Di Pietro, A.; Walton, J. D.; Ma, L. J.; Baker, S. E.; Rep, M., et al., The Fusarium graminearum genome reveals a link between localized polymorphism and pathogen specialization. *Science (New York, N.Y.)* 2007, *317* (5843), 1400-1402.

2. Zheng, Q.; Yu, Z.; Yuan, Y.; Sun, D.; Abubakar, Y. S.; Zhou, J.; Wang, Z.; Zheng, H., The GTPase-Activating Protein FgGyp1 Is Important for Vegetative Growth, Conidiation, and Virulence and Negatively Regulates DON Biosynthesis in Fusarium graminearium. *Frontiers in microbiology* 2021, *12*, 621519.

3. Zheng, H.; Li, L.; Yu, Z.; Yuan, Y.; Zheng, Q.; Xie, Q.; Li, G.; Abubakar, Y. S.; Zhou, J.; Wang, Z., et al., FgSpa2 recruits FgMsb3, a Rab8 GAP, to the polarisome to regulate polarized trafficking, growth and pathogenicity in Fusarium graminearum. *New Phytol* 2021, *229* (3), 1665-1683.
